# Complications Associated with Enteral Nutrition: CAFANE Study

**DOI:** 10.3390/nu11092041

**Published:** 2019-09-01

**Authors:** Carmina Wanden-Berghe, Maria-Carmen Patino-Alonso, Purificación Galindo-Villardón, Javier Sanz-Valero

**Affiliations:** 1 Grupo de Nutrición Clínica y HAD del Instituto de Investigación Sanitaria y Biomédica de Alicante (ISABIAL—Fundación FISABIO), Hospital General Universitario de Alicante, 03010 Alicante, Spain; 2 Departamento de Estadística, Universidad de Salamanca, 37007 Salamanca, Spain; 3 Departamento Salud Pública & Historia de la Ciencia, Campus de Sant Joan, Universidad Miguel Hernández, 03550 Alicante, Spain

**Keywords:** enteral nutrition, nutritional support, complications, administration

## Abstract

Objectives: To determine the association between home enteral nutrition (HEN) administration modality and its complications in patients. Methods: This is a prospective multicenter longitudinal study including 15 Spanish hospitals, from April 2015 to March 2017. A 4-month follow-up period was conducted for each patient by home visit. The study subjects were adult patients who began their nutrient intake by tube feeding, known as HEN, during the recruitment period. The variables studied included the type and modality of HEN administration and its related complications, such as vomiting, regurgitation, constipation, diarrhea, and abdominal distention. Mechanical complications and bronchoaspiration were also evaluated. Descriptive variables were used for fitting. Results: The study consisted of 306 patients; 4 were lost due to death. Specific HEN modalities protected against constipation (odds ratio (OR) = 0.4) and regurgitation (OR = 0.4). The use of a nasogastric tube (NGT) resulted in a lower risk of diarrhea compared to percutaneous endoscopic gastrostomy (PEG) (OR = 0.4) but resulted in a higher risk of tube obstruction (OR = 7.4). The use of intermittent gravity versus bolus feeding was a protection factor against vomiting (OR = 0.4), regurgitation (OR = 0.3), constipation (OR = 0.3), diarrhea (OR = 0.4) and abdominal distension (OR = 0.4). The increase in the number of doses was a risk factor for the incidence of regurgitation (OR = 1.3). Conclusions: Gastrointestinal complications were the most frequent problems, but an adequate choice of the formula, route, feeding modality, number of doses, administration time, and dose volume can reduce the risk of these complications.

## 1. Introduction

Keeping a patient hospitalized for the sole purpose of administering nutritional support has become an inappropriate decision from a bio-psycho-social perspective, and wasteful for health institutions and for society from an economic perspective. The introduction of home enteral nutrition (HEN) involves the concerns of correctly selecting candidates, applying a good training programme to the patient and the caregiver, ensuring the supply of formula and necessary materials, providing adequate follow-up and monitoring the quality of patient care [1].

Interestingly, Parsons et al. [2] concluded that for patients admitted to nursing homes, oral nutritional support could improve the quality of life and nutrient intake more effectively than dietary advice.

In Spain, HEN is included and regulated in the portfolio of services of the National Health System [3]. This legislative norm, along with the benefits of HEN itself and the development of nutrition units and home hospitalization units [4], have made this method of nutritional support (NS) the primary choice for patients who are malnourished or are at risk of malnutrition and have preserved digestive system function but cannot meet their nutritional requirements by natural nutrition alone [5].

HEN is a safe procedure [6] whose complications can be predicted and controlled by protocols that consider the formula prescription, the route of administration, the care, the selection of the formula to be used and the feeding modality [7]. In addition, NS helps improve the patient’s quality of life [8,9].

Knowing the HEN-related complications is crucial to achieving the objectives of this therapy and to determining its safety level outside the hospital setting, which justifies this study. For the nutritional treatment to be carried out effectively and safely, it is essential that the patient and/or the main caregiver acquires a degree of responsibility in managing the administration of nutrition and, above all, can detect the first signs or symptoms of possible complications. Caregivers must provide the first point of care and demand proper healthcare services for the patient.

The different types of complications and possible medical interventions associated with HEN are already known [7,8,9,10]. However, the elements that can influence the incidence of HEN-related complications have not been determined. Consequently, due to the limited understanding of enteral formula administration methods and their association with complications, this study aims to determine the association between HEN administration modality and the complications presented by patients.

## 2. Methods

### 2.1. Study Design

This is a prospective and multicenter longitudinal study.

### 2.2. Setting

The study involved 15 Spanish hospitals from 7 different autonomous communities: Valencia, Madrid, Cantabria, Canarias, Andalucía, Aragón, and Baleares. Recruitment was carried out from April 2015 to March 2017. The data collection and follow-up period for the patients included in the study was 4 months and was conducted through home visits.

### 2.3. Participants

Inclusion criteria: Adult patients (≥18 years) who were admitted to the participating home hospitalization units or were dependent on nutrition units, who began their nutrient intake by tube feeding, known as home enteral nutrition (HEN), during the recruitment period.

During the four month follow-up a monthly visit was carried out by the head of each of the participating hospitals, moment in which the patient or their caregiver reported on the adverse effects incurred.

All patients had to provide informed consent to be included in the study. In cases where the patient’s situation did not allow for this, consent was obtained from the primary caregiver.

### 2.4. Ethical Requirements

The protocol used in this work was approved by the Clinical Research Ethics Committee of the General University Hospital of Alicante (Hospital General Universitario de Alicante), dated January 8, 2014 (registration number CE20PI2013/40).

### 2.5. Data Collection

The data logging was performed through a platform available on the Internet (http://www.cafane.net/), which provided an ad hoc questionnaire developed for this study. The questionnaire complied with all data protection regulations; therefore, it did not record any patient personal data. The participating researchers were granted personalized access (username and password). The questionnaire variables are listed below.

Variables related to the type and modality of HEN administration.

Type of formula administered: standard, specific and others (hypercaloric, hyperproteic, hypercaloric-hyperproteic); HEN with fiber: yes or no; Route of administration: percutaneous endoscopic gastrostomy (PEG), nasogastric tube (NGT) and other ostomies (including jejunostomy); Feeding modality: bolus feeding, intermittent gravity, other modalities (including continuous gravity or pump feeding); Administration time of each intake in minutes; Number of intake periods per day; Dose volume (mL); Total daily volume (mL); Washing of the probe: yes or no; Position during intake (≥45° or <45°) and after intake (at least 1 h) [11].

### 2.6. HEN-Related Complications

Digestive complications: vomiting, regurgitation, constipation, diarrhea and abdominal distention; Mechanical complications: tube obstruction; Other complications: aspiration pneumonia (bronchoaspiration).

### 2.7. Descriptive and Fitting Variables

Gender: man or woman; Age: in years; Weight: in kilograms (kg) (if the weight could not be obtained by direct measurement, it was estimated according to gender, age, arm circumference and knee height using the “Malnutrition Universal Screening Tool” [12]); Height: in meters (m) (if the height could not be obtained, it was estimated by the length of the forearm [12]); Body mass index (BMI): weight (kg) divided by the height squared (m); Place of residence: family home or social-health institution; Caregiver: family member, paid family member, employee, volunteer or without caregiver; Diagnostic groups: neurological, oncological, and with other diagnoses.

### 2.8. Statistical Analysis

For the qualitative variables, the absolute and relative frequencies (percentages) were calculated. For the quantitative variables, the mean and its standard deviation, the median (Me), the interquartile range (IQR) and the standard deviation were calculated.

The association between the qualitative variables was analyzeanalyzed by the chi-squared test, while Student’s t-test was used for the quantitative variables to verify the significance of the difference in means for the independent samples. To compare the means between more than 2 groups for a quantitative variable, analysis of variance (ANOVA) was performed using the Tukey method.

The incidence rate (IR) was calculated to find the number of complications involved in the study period (4 months).

A logistic regression model was applied using each of the different complications as a dependent variable (0 = no complication, 1 = one or more complications). The independent variables were route of administration (1 = PEG, 2 = NGT; 3 = other ostomies); feeding modality (1 = intermittent gravity, 2 = bolus feeding, 3 = other modalities); fiber (1 = yes; 2 = no); administration time; number of intake periods; volume of intake; and total volume. All models included the following variables: gender, age, BMI, place of residence, caregiver, and diagnostic group. The probability was measured by the odds ratio (OR).

The level of significance used in all hypotheses was *p* ≤ 0.05.

Quality control of the data was carried out through double tables, and the potential errors were corrected by consulting the originals. The statistical software IBM SPSS Statistics for Windows, version 23.0 (IBM Corp, Armonk, NY, USA) was used to analyze the data.

## 3. Results

The data from 306 patients were included; 4 patients were lost due to death. The descriptive data of the population is presented in Table 1. The results show that the place of residence was primarily the family home (224 patients, 73.2%). With the exception of 16 patients (5.2%), most had some type of caregiver, generally a relative (226 patients, 73.9%), and this person was usually a woman (246 cases, 80.4%).

The base pathology did not influence the relationship of the complications associated with the HEN modality.

No intergroup associations were found between the BMI and the mean of the majority of the complications: vomiting (*p* = 0.054), regurgitation (*p* = 0.415), constipation (*p* = 0.401), diarrhea (*p* = 0.113) and probe obstruction (0.204). However, there were significant differences in abdominal distension between the normal weight and obese groups (*p* = 0.040) and for aspiration pneumonia among the overweight and obese groups (*p* = 0.031).

Regarding the HEN-related variables, all of the patients were tube-fed, with 254 (83.0%) patients being exclusively tube-fed, while 52 (17.0%) took in some food orally. The HEN-related descriptive results and the HEN administration modalities are presented in Table 2.

The most frequent complications recorded in the 4 months of follow-up were digestive, predominantly abdominal distension, with an IR of 2.4, while regurgitation had an IR of 2.2. Aspiration pneumonia presented the lowest IR of 0.1. Some significant differences were found, with higher values in men for abdominal distension (3.5 versus 1.1, *p*-value 0.001) and constipation (3.5 versus 1.1, *p*-value 0.001). The number of episodes (*n*) and the IRs of HEN-related complications are shown in Table 3.

## 4. Complications Related to the HEN Type and Modality

When analyzing the pre- and post-fitting results regarding the relation between complications and the type of formula used (see Table 4), it was found that the use of a specific HEN formula protected against regurgitation episodes (OR = 0.5 in pre-fitting; OR = 0.4 in post-fitting) and constipation (OR = 0.5 in pre-fitting; OR = 0.4 in post-fitting). On the other hand, the use of other formulas (hypercaloric, hyperproteic, hypercaloric-hyperproteic) was associated with a greater incidence of aspiration pneumonia (OR = 2.4 in pre-fitting; OR = 2.7 in post-fitting).

The use of NGT presented less risk of diarrhea compared to PEG (OR = 0.3 in pre-fitting; OR = 0.4 in post-fitting). Likewise, in the post-fitting results, NGT showed a lower risk of vomiting (OR = 0.4), regurgitation (OR = 0.4) and abdominal distension (OR = 0.5). However, a greater risk of probe obstruction was observed (OR = 7.4).

Regarding the feeding modality, the use of intermittent gravity was a protective factor against vomiting (OR = 0.4, in pre-fitting and post-fitting), regurgitation (OR = 0.3, in pre-fitting and post-fitting), constipation (OR = 0.4 in pre-fitting; OR = 0.3 in post-fitting), diarrhea (OR = 0.5 in pre-fitting; OR = 0.4 in post-fitting) and abdominal distension (OR = 0.3 in pre-fitting; OR = 0.4 in post-fitting) when compared to bolus feeding. However, in the case of abdominal distension, other feeding modalities were also more favourable than bolus feeding (OR = 0.2 in pre-fitting; OR = 0.1 in post-fitting).

The use of HEN with fiber presented a lower risk of constipation (OR = 0.2, in pre-fitting and post-fitting).

The increase in the number of doses represented a risk for the appearance of regurgitation (OR = 1.3 in pre-fitting and post-fitting) and for the obstruction of the catheter (OR = 1.9 in pre-fitting; OR = 1.7 in post-fitting). Likewise, the intake position was associated with probe obstruction (OR = 0.1 in pre-fitting and post-fitting).

The post-fitting results showed association with an increased risk of diarrhea when the administration time (OR = 4.5) and the dose volume (OR = 1.4) increased.

The results of the complications related to the type and modality of HEN administration are shown in Table 4.

## 5. Discussion

This study enabled us to determine the complications associated with enteral nutrition administration in a considerable number of patients over a period of several months, which is a strength of this study.

In terms of their pathology, the studied patients presented similar characteristics as those of previous studies—they were older adults with neurological or oncological disease, with a dependence on and a need for a caregiver [13,14].

There was no clear significant association between the BMI and the complications resulting from HEN, although a greater number of complication episodes in relation to obesity was observed, a situation already highlighted by Wiggins et al. [15].

Given the median age of the studied population, it is normal in the Spanish socio-cultural context that the place of residence was primarily the family home, a nucleus with sufficient roots and a caretaker tradition. Likewise, the fact that the main caregiver is typically a woman has been widely noted in the scientific literature [16,17] and must be considered when implementing an artificial nutrition regimen, since it can be a great burden for the caregiver [18].

The greater use of PEG compared to NGT, common in the participating health centers, contrasts with previous research that observed a greater tendency to use NGT in older adults [1,14,19]. In any case, gastrostomy is associated with greater efficacy and safety compared to NGT [20].

Bolus feeding continued to be used with a greater frequency, though it could be inferred that the infusion speed is not easy to regulate, and there could be alterations in the administration that would lead to some complications. On the other hand, the formula type, the volume administered and the number of doses were all within normal ranges, and it was not surprising that a formula with fiber was administered to patients with long-term HEN, a recommended practice in the absence of contraindicateons [21].

The IR of HEN-related complications demonstrated the intimate relationship with the route and administration modality. The correct management of HEN reduces these complications and minimizes laryngopharyngeal reflux [22].

The differences in the observed complications between men and women show an important gender background. Women typically take better responsibility for their own healthcare, especially regarding communication between the patient and the doctor, the understanding of the disease, and their attitudes at the end of life [23,24].

## 6. Complications Related to the Type and Modality of HEN Administration

It is important to know that specific formulas are associated with fewer episodes of regurgitation and constipation. However, in many patients, these complications are already present before the initiation of HEN therapy. Therefore, the addition of prokinetic drugs would be useful to prevent regurgitation. In addition, constipation is more frequent than diarrhea in patients fed exclusively by HEN [25].

The current evidence does not allow us to determine the causes of the increased risk of aspiration pneumonia in nonspecific HEN; the evidence could possibly indicate a recommendation for using a post-pyloric probe, but it is not certain [26].

Regarding the administration modality, PEG has become the method of choice for enteral feeding in the medium and long term. Most of the complications related to PEG are minor; however, rare major complications can be more serious. The increased risk of diarrhea, vomiting and regurgitation that was observed in this study in relation to PEG was mainly associated with abdominal distension and could show a stronger relation with the administration modality and the volume of the shot. Some complications occur shortly after tube placement; others develop later when the gastrostomy tract has matured. Senior patients with comorbidities and infections appear to be at greater risk of developing complications [27]. Gomes et al. [20] demonstrated the nonexistence of significant differences in mortality rates or adverse events, including aspiration pneumonia, between comparison groups (PEG versus NGT). However, they pointed out that factors such as the demographics of the participants, the underlying diseases, age, gender and the gastrostomy technique should be considered. In this sense, Gomez-Candela et al. [19] indicated that to achieve a low incidence of complications, it was essential to establish an adequate educational programme.

Bolus feeding has been associated with virtually all gastrointestinal complications, and this feeding modality may explain the increased infusion rate, with an abrupt change in the gastrointestinal walls or rapid temperature change. Contrary to these findings, a previous study by Kadamani et al. [28] did not find differences in the incidence of complications between both administration modalities. However, according to scientific evidence, continuous nutrition should always be chosen for infants with birth weights below 1250 g or infants with haemodynamic deterioration [29].

Likewise, the present study has shown that an enteral diet containing fiber is a protective factor against intestinal motility disorders [25,30].

It has also become clear that an increase in the number of doses and the volume of the intake cause greater gastrointestinal problems and that the patient’s position upon intake is related to the possibility of probe obstruction.

## 7. Conclusions

It can be concluded that there was a higher incidence of gastrointestinal complications. However, an adequate choice of the formula type, the route and feeding modality, the number of doses, administration time and volume of intake can greatly reduce the IR. Therefore, to reduce these complications, the existence of multidisciplinary teams focused on the follow-up of patients is essential to optimize the results. However, all health care providers should have knowledge regarding the most frequent HEN-related complications and the skills to manage these problems.

## Figures and Tables

**Table 1:** Descriptive data related to the study population.

Variables	Men	Women	*p*-Value
Number of patients	168 (54.9%)	138 (45.1%)	
Age (years)			
Mean	70.2 ± 1.0	71.9 ± 1.4	0.330
Median	72.0	77.0	
IQR ^1^	61.3–80.0	63.5–84.0	
Standard deviation	12.9	17.1	
Maximum/Minimum	91.0/36.0	94.0/18.0	
Weight (kg)			
Mean	63.9 ± 0.9	56.4 ± 1.1	<0.001
Median	64.0	55.0	
IQR ^1^	57.0–72.0	47.9–65.0	
Standard deviation	11.5	12.5	
Maximum/Minimum	86.0/36.0	85.0/26.5	
Height (m)			
Mean	1.7 ± 0.1	1.6 ± 0.0	<0.001
Median	1.7	1.6	
IQR ^1^	1.7–1.8	1.6–1.6	
Standard deviation	0.1	0.1	
Maximum/Minimum	2.1/1.5	1.8/1.4	
Body mass index (kg/m^2^)			
Mean	26.1 ± 0.4	27.1 ± 0.5	0.102
Median	25.2	27.3	
IQR ^1^	22.6–29.7	22.1–31.4	
Standard deviation	4.5	6.1	
Maximum/Minimum	35.5/16.4	42.2/15.0	
Place of residence			
Family address	148 (48.4%)	90 (29.4%)	<0.001
Socio-sanitary institution	20 (6.5%)	48 (15.7%)	
Care provider			
Family	136 (44.4%)	88 (28.8%)	<0.001
Paid family member	2 (0.7%)	-	
Employee	14 (4.6%)	40 (13.1%)	
Voluntary	-	10 (3.3%)	
Without caregiver	16 (5.2%)	-	
Diagnosis			
Neurological	86 (28.1%)	108 (35.3%)	
Oncology	66 (21.6%)	14 (4.6%)	<0.001
Other diagnoses	16 (5.2%)	16 (5.2%)	

^1^ IQR = Interquartile Range.

**Table 2:** Descriptive results related to HEN and its administration.

Variables	Men	Women	*p*-Value
Number of patients	168 (54.9%)	138 (45.1%)	
Formula type			
Standard	56 (18.3%)	50 (16.3%)	
Specific Other	60 (19.6%)	42 (13.7%)	0.003
	52 (17.0%)	46 (15.1%)	
Fiber content			
Yes	156 (50.9%)	132 (43.1%)	0.301
No	12 (3.9%)	6 (2.0%)	
Route of administration			
NGT ^1^	38 (12.4%)	48 (15.7%)	
PEG ^2^ Other ostomies	118 (38.6%)	88 (29.0%)	0.018
	12 (3.9%)	2 (0.7%)	
Method of administration			
Bolus	102 (33.3%)	68 (22.2%)	0.128
Intermittent gravity Other		52 (17.0%)	55 18.0%)
		14 (4.6%)	15 (4.9%)
Administration time (min)			
Mean	39.2 ± 5.6	44.3 ± 6.6	0.557
Median	30.0	30.0	
IQR ^3^	20.0–40.0	15.0–41.3	
Standard deviation	72.3	77.0	
Maximum/Minimum	720.0/3.0	780.0/3.0	
Number of intake periods per day			
Mean	4.4 ± 0.1	4.2 ± 0.1	0.062
Median	5.0	4.0	
IQR ^3^	4.0–5.0	3.0–5.0	
Standard deviation	1.2	1.1	
Maximum/Minimum	9.0/1.0	6.0/1.0	
Volume of the shot (mL)			
Mean	301.5 ± 10.0	338.6 ± 14.4	0.035
Median	290.0	300.0	
IQR ^3^	200.0–350.0	250.0–400.0	
Standard deviation	129.6	168.7	
Maximum/Minimum	1000.0/100.0	1250.0/200.0	
Total daily volume (mL)			
Mean	1644.9 ± 41.8	1686.0 ± 33.2	0.442
Median	1535.0	1700.0	
IQR ^3^	1275.0–2000.0	1500.0–1800.0	
Standard deviation	542.1	390.1	
Maximum/Minimum	3400.0/700.0	3500.0/700.0	
Probe washing (pre and post)			
Yes	143 (46.7%)	120 (39.2%)	
No	25 (8.2%)	18 (5.9%)	
Position during and after intake			
Less than 45°	148 (48.4%)	132 (43.1%)	0.018
Equal or more than 45°	20 (6.5%)	6 (2.0%)	

^1^ NGT = nasogastric tube. ^2^ PEG = endoscopic percutaneous gastrostomy. ^3^ IQR = interquartile range.

**Table 3:** Number of episodes (*n*) and incidence rate (IR) of HEN-related complications.

Variables	Men	Women	*p*-Value
	n/IR	n/IR	
Digestive complications			
Vomiting	204/1.2	124/0.9	0.571
Regurgitation	433/2.6	236/1.7	0.304
Constipation	318/1.9	154/1.1	0.024
DiarrheaDiarrhea	270/1.6	84/0.6	0.004
Abdominal distension	582/3.5	146/1.1	0.001
Mechanical complications of the probe			
Obstruction	110/0.6	14/0.1	<0.001
Other complications			
Aspiration pneumonia	24/0.1	20/0.1	0.967

**Table 4:** Complications related to the type and modality of HEN administration.

Complication	HEN-related Variables	Pre-Fitting	*p*-Value	Post-Fitting	*p*-Value
Vomiting	Formula type: Standard				
	Specific	0.6 (0.3–1.4)	0.611	0.6 (0.2–1.4)	0.230
	Other ^a^	1.5 (0.7–3.1)	1.489	1.4 (0.6–3.1)	0.473
	Administration route: PEG ^b^				
	NGT ^b^	0.6 (0.3–1.2)	0.118	0.4 (0.2–0.9)	0.026
	Other ^c^	1.8 (0.5–6.3)	0.392	0.8 (0.2–3.3)	0.722
	Administration modality: Bolus				
	Intermittent gravity	0.4 (0.2–0.9)	0.021	0.4 (0.2–0.9)	0.037
	Other ^d^				
	Fiber ^e^	3.3 (1.2–9.3)	0.025	2.6 (0.8–7.9)	0.100
	Administration time	1.0 (1.0–1.0)	0.222	1.0 (1.0–1.0)	0.089
	Number of intake periods per day	0.8 (0.6–1.1)	0.106	0.8 (0.6–1.1)	0.151
	Volume of the intake	1.0 (1.0–1.0)	0.948	1.0 (1.0–1.0)	0.818
	Total daily volume	1.0 (1.0–1.0)	0.003	1.0 (1.0–1.0)	0.026
	Probe washing ^f^	1.4 (0.6–3.4)	0.396	1.4 (0.6–3.4)	0.440
	Position ^g^	1.9 (0.7–5.1)	0.193	1.4 (0.5–4.1)	0.492
Regurgitation	Formula type: Standard				
	Specific	0.5 (0.2–1.0)	0.041	0.4 (0.2–1.0)	0.037
	Other ^a^	1.2 (0.6–2.3)	0.635	1.0 (0.5–2.1)	0.992
	Route of administration: PEG ^b^				
	NGT ^b^	0.7 (0.4–1.4)	0.349	0.4 (0.2–0.9)	0.031
	Other ^c^	-	0.999	-	0.998
	Administration modality: Bolus				
	Intermittent gravity	0.3 (0.2–0.7)	0.002	0.3 (0.1–0.6)	0.002
	Other ^d^	-	0.998	-	0.908
	Fiber ^e^	-	-	-	-
	Administration time	1.0 (1.0–1.0)	0.085	1.0 (1.0–1.0)	0.122
	Number of intake periods per day	1.3 (1.0–1.6)	0.064	1.3 (1.0–1.7)	0.039
	Volume of the intake	1.0 (1.0–1.0)	0.015	1.0 (1.0–1.0)	0.019
	Total daily volume	1.0 (1.0–1.0)	0.250	1.0 (1.0–1.0)	0.683
	Probe washing ^f^	1.4 (0.6–3.0)	0.439	1.3 (0.6–3.1)	0.493
	Position ^g^	2.0 (0.8–5.0)	0.114	0.8 (0.4–1.6)	0.470
Constipation	Formula type: Standard				
	Specific	0.5 (0.3–0.8)	0.012	0.4 (0.2–0.8)	0.006
	Other ^a^	1.4 (0.8–2.6)	0.221	1.4 (0.7–2.6)	0.343
	Route of administration: PEG ^b^				
	NGT ^b^	0.8 (0.5–1.4)	0.832	0.6 (0.3–1.1)	0.079
	Other ^c^	0.3 (0.1–1.3)	0.281	0.1 (0.0–0.6)	0.013
	Administration modality: Bolus				
	Intermittent gravity	0.4 (0.2–0.7)	0.001	0.3 (0.2–0.6)	0.000
	Other ^d^	0.6 (0.3–1.5)	0.305	0.7 (0.3–1.8)	0.474
	Fiber ^e^	0.2 (0.1–1.0)	0.057	0.2 (0.0–0.8)	0.027
	Administration time	1.0 (1.0–1.0)	0.082	1.6 (0.6–4.2)	0.367
	Number of intake periods per day	0.8 (0.7–1.0)	0.058	1.0 (1.0–1.0)	0.076
	Volume of the intake	1.0 (1.0–1.0)	0.479	0.8 (0.7–1.0)	0.072
	Total daily volume	1.0 (1.0–1.0)	0.036	1.0 (1.0–1.0)	0.683
	Probe washing ^f^	0.7 (0.4–1.5)	0.417	1.0 (1.0–1.0)	0.030
	Position ^g^	3.6 (1.6–8.3)	0.002	1.7 (0.8–3.4)	0.165
Diarrhea	Formula type: Standard				
	Specific	0.7 (0.4–1.3)	0.295	0.6 (0.3–1.2)	0.159
	Other ^a^	0.7 (0.4–1.4)	0.326	0.6 (0.3–1.3)	0.192
	Route of administration: PEG ^b^				
	NGT ^b^	0.3 (0.2–0.5)	0.000	0.4 (0.2–0.7)	0.006
	Other ^c^	1.9 (0.6–5.8)	0.290	0.1 (0.1–0.7)	0.017
	Administration modality: Bolus				
	Intermittent gravity	0.5 (0.3–0.8)	0.011	0.4 (0.2–0.8)	0.007
	Other ^d^	0.5 (0.2–1.4)	0.193	0.5 (0.2–1.3)	0.165
	Fiber ^e^	1.4 (0.5–3.9)	0.520	1.2 (0.4–3.3)	0.760
	Administration time	1.0 (1.0–1.0)	0.690	4.5 (1.9–11.0)	0.001
	Number of intake periods per day	1.4 (1.1–1.7)	0.010	1.0 (1.0–1.0)	0.659
	Volume of the intake	1.0 (1.0–1.0)	0.179	1.4 (1.1–1.7)	0.011
	Total daily volume	1.0 (1.0–1.0)	0.429	1.0 (1.0–1.0)	0.301
	Probe washing ^f^	1.6 (0.8–3.1)	0.199	0.9 (0.4–2.0)	0.801
	Position ^g^	1.8 (0.8–4.2)	0.165	1.7 (0.7–4.0)	0.244
Abdominal distension	Formula type: Standard				
	Specific	1.3 (0.7–2.7)	0.402	1.0 (0.6–2.6)	0.551
	Other ^a^	2.6 (1.3–5.2)	0.005	2.1 (1.0–4.4)	0.053
	Route of administration: PEG ^b^				
	NGT ^b^	0.8 (0.4–1.4)	0.432	0.5 (0.3–0.9)	0.049
	Other ^c^	0.2 (0.3–4.1)	0.813	0.2 (0.1–0.9)	0.043
	Administration modality: Bolus				
	Intermittent gravity	0.3 (0.2–0.6)	0.001	0.4 (0.2–0.7)	0.006
	Other ^d^	0.2 (0.1–0.7)	0.014	0.1 (0.1–0.7)	0.017
	Fiber ^e^	0.4 (0.1–1.8)	0.235	0.2 (0.0–0.9)	0.038
	Administration time	0.3 (1.0–1.0)	0.323	1.0 (1.0–1.0)	0.414
	Number of intake periods per day	1.1 (1.0–1.4)	0.269	1.1 (0.9–1.4)	0.298
	Volume of the intake	1.0 (1.0–1.0)	0.012	1.0 (1.0–1.0)	0.171
	Total daily volume	1.0 (1.0–1.0)	0.018	1.0 (1.0–1.0)	0.104
	Probe washing ^f^	1.0 (0.5–2.2)	0.949	0.4 (1.0–1.4)	0.150
	Position ^g^	1.6 (0.6–3.8)	0.320	1.1 (0.4–2.7)	1.057
Obstruction of the probe	Formula type: Standard				
	Specific	0.6 (0.2–2.3)	0.481	0.7 (0.2–2.7)	0.555
	Other ^a^	0.5 (0.1–1.9)	0.328	0.5 (0.1–2.3)	0.400
	Route of administration: PEG ^b^				
	NGT ^b^	1.9 (0.6–5.5)	0.266	7.4 (1.6–33.8)	0.010
	Other ^c^	0.5 (0.1–2.5)	0.360	5.9 (0.7–50.7)	0.107
	Administration modality: Bolus				
	Intermittent gravity	4.7 (1.0–21.2)	0.043	2.3 (0.4–12.7)	0.317
	Other ^d^	-	0.998	-	0.998
	Fiber ^e^	0.4 (0.1–2.0)	0.263	0.9 (0.2–5.0)	0.897
	Administration time	1.0 (1.0–1.0)	0.700	1.0 (1.0–1.0)	0.832
	Number of intake periods per day	1.9 (1.3–2.8)	0.002	1.7 (1.1–2.5)	0.020
	Volume of the intake	1.0 (1.0–1.0)	0.568	1.0 (1.0–1.0)	0.024
	Total daily volume	1.0 (1.0–1.0)	0.000	1.0 (1.0–1.0)	0.896
	Probe washing ^f^	0.5 (0.1–1.5)	0.205	1.0 (1.0–1.0)	0.028
	Position ^g^	0.1 (0.0–0.4)	0.000	0.1 (0.0–0.5)	0.005
Aspiration pneumonia	Formula type: Standard				
	Specific	2.0 (0.8–4.9)	0.153	2.1 (0.8–5.4)	0.132
	Other ^a^	2.4 (1.0–6.0)	0.065	2.7 (1.0–7.5)	0.054
	Route of administration: PEG ^b^				
	NGT ^b^	0.6 (0.3–1.3)	0.188	0.5 (0.2–1.2)	0.123
	Other ^c^	-	0.999	-	0.996
	Administration modality: Bolus				
	Intermittent gravity	1.1 (0.5–2.3)	0.745	0.8 (0.3–1.7)	0.532
	Other ^d^	0.6 (0.1–2.5)	0.446	0.9 (0.2–5.1)	0.996
	Fiber ^e^	0.9 (0.2–4.2)	0.929	1.0 (0.2–4.8)	0.977
	Administration time	1.0 (1.0–1.0)	0.098	1.0 (1.0–1.0)	0.204
	Number of intake periods per day	1.0 (0.8–1.5)	0.597	1.0 (0.8–1.4)	0.849
	Volume of the intake	1.0 (1.0–1.0)	0.718	1.0 (1.0–1.0)	0.790
	Total daily volume	1.0 (1.0–1.0)	0.983	1.0 (1.0–1.0)	0.934
	Probe washing ^f^	1.9 (0.8–4.5)	0.139	2.0 (0.8–4.8)	0.148

^a^ Other (hypercaloric, hyperproteic, hypercaloric-hyperproteic); ^b^ PEG = percutaneous endoscopic gastrostomy; NGT = nasogastric tube; ^c^ Other ostomies (including jejunostomy); ^d^ Other forms (including continuous gravity or pump feeding); ^e^ Fiber (yes/no); ^f^ Washing (yes/no); ^g^ position (position during intake, ≥45° or <45° and maintained at least 1 h after intake).
